# Comparative Analysis of Root Microbiomes of Rice Cultivars with High and Low Methane Emissions Reveals Differences in Abundance of Methanogenic Archaea and Putative Upstream Fermenters

**DOI:** 10.1128/mSystems.00897-19

**Published:** 2020-02-18

**Authors:** Zachary Liechty, Christian Santos-Medellín, Joseph Edwards, Bao Nguyen, David Mikhail, Shane Eason, Gregory Phillips, Venkatesan Sundaresan

**Affiliations:** a Department of Plant Biology, University of California, Davis, Davis, California, USA; b Department of Agriculture, Arkansas State University, Jonesboro, Arkansas, USA; c Department of Plant Sciences, University of California, Davis, Davis, California, USA; University of Tennessee at Knoxville

**Keywords:** endosphere, fermentation, methane, methanogenesis, microbiome, rhizoplane, rhizosphere, rice, root

## Abstract

Rice cultivation worldwide accounts for ∼7 to 17% of global methane emissions. Methane cycling in rice paddies is a microbial process not only involving methane producers (methanogens) and methane metabolizers (methanotrophs) but also other microbial taxa that affect upstream processes related to methane metabolism. Rice cultivars vary in their rates of methane emissions, but the influence of rice genotypes on methane cycling microbiota has been poorly characterized. Here, we profiled the rhizosphere, rhizoplane, and endosphere microbiomes of a high-methane-emitting cultivar (Sabine) and a low-methane-emitting cultivar (CLXL745) throughout the growing season to identify variations in the archaeal and bacterial communities relating to methane emissions. The rhizosphere of the high-emitting cultivar was enriched in methanogens compared to that in the low emitter, whereas the relative abundances of methanotrophs between the cultivars were not significantly different. Further analysis of cultivar-sensitive taxa identified families enriched in the high emitter that are associated with methanogenesis-related processes. The high emitter had greater relative abundances of sulfate-reducing and iron-reducing taxa which peak earlier in the season than methanogens and are necessary to lower soil oxidation reduction potential before methanogenesis can occur. The high emitter also had a greater abundance of fermentative taxa which produce methanogenesis precursors (acetate, CO_2_, and H_2_). Furthermore, the high emitter was enriched in taxa related to acetogenesis which compete with methanogens for CO_2_ and H_2_. These taxa were enriched in a spatio-specific manner and reveal a complex network of microbial interactions on which plant genotype-dependent factors can act to affect methanogenesis and methane emissions.

**IMPORTANCE** Rice cultivation is a major source of anthropogenic emissions of methane, a greenhouse gas with a potentially severe impact on climate change. Emission variation between rice cultivars suggests the feasibility of breeding low-emission rice, but there is a limited understanding of how genotypes affect the microbiota involved in methane cycling. Here, we show that the root microbiome of the high-emitting cultivar is enriched both in methanogens and in taxa associated with fermentation, iron, and sulfate reduction and acetogenesis, processes that support methanogenesis. Understanding how cultivars affect microbes with methanogenesis-related functions is vital for understanding the genetic basis for methane emission in rice and can aid in the development of breeding programs that reduce the environmental impact of rice cultivation.

## Abstract (precis)

Rice cultivation is a major source of anthropogenic emissions of methane, a greenhouse gas with a potentially severe impact on climate change. Emission variation between rice cultivars suggests the feasibility of breeding low-emission rice, but there is a limited understanding of how genotypes affect the microbiota involved in methane cycling. Here, we show that the root microbiome of the high-emitting cultivar is enriched both in methanogens and in taxa associated with fermentation, iron, and sulfate reduction and acetogenesis, processes that support methanogenesis. Understanding how cultivars affect microbes with methanogenesis-related functions is vital for understanding the genetic basis for methane emission in rice and can aid in the development of breeding programs that reduce the environmental impact of rice cultivation.

## INTRODUCTION

Methane (CH_4_), a potent greenhouse gas, has 28 times the global warming potential of CO_2_ (1). A major source of anthropogenic CH_4_ emissions is rice cultivation, which accounts for approximately 7 to 17% of the global CH_4_ sources (25 to 100 terragrams [Tg] of CH_4_ per year) (2–4). Methane is produced by facultative anaerobic archaea in the rice rhizosphere, which subsist predominantly on carbon sources originating from the rice plant, such as root exudate (5, 6). After production in the rhizosphere, CH_4_ diffuses into the root airspaces (aerenchyma) and is transported through the plant and into the atmosphere. Up to 80% of the CH_4_ produced in soils of paddy fields was found to be transported into the atmosphere through the aerenchyma of rice plants (7). Methanogens interact positively and negatively with several microbial taxa that influence the rate of methanogenesis. Methanogens cannot directly consume complex root exudates but rather rely on the fermentative activity of syntrophic microbes to produce methanogenic precursor molecules such as acetate, H_2_, and CO_2_ (8, 9). On the other hand, methanotrophic bacteria oxidize CH_4_ and reduce the amount of CH_4_ emitted by up to 60% (10). Methanogens can also be outcompeted by microbes that consume the same precursor molecules, such as anaerobic respiring microbes that reduce nitrate, sulfate, and iron (9, 11).

An effort to mitigate the environmental impact of rice cultivation using a transgenic approach has been reported (12). An alternate approach is to exploit natural variation in CH_4_ emissions between rice genotypes. A survey of different rice cultivars identified varieties that exhibit divergence in CH_4_ emissions through the growing season, with up to 2-fold variation in average seasonal CH_4_ emissions between the high- and low-emitting cultivars (13). Understanding the underlying causes behind these genotype-mediated differences in CH_4_ emissions could lead to mitigation strategies to curb the environmental cost of rice cultivation. Genotypic variation has been shown to directly affect the microbial composition of methanogens and methanotrophs, and low emitters have been reported to have an increased abundance of methanotrophs (14, 15). However, these studies were limited to only estimating methanogen and methanotrophs and did not survey the compositional profiles of all bacteria and archaea in the root microbiomes. By profiling full bacterial and archaeal communities, we can identify variations in the abundances not only of methanogens and methanotrophs but also in other microbes fulfilling the above-mentioned niches related to methanogenesis. We have previously demonstrated that the rice root microbiome exhibits a reproducible dependence on plant genotype (16, 17). Rice microbiomes are also spatially structured in compositionally distinct compartments, namely, the rhizosphere (soil directly influenced by root activity), the rhizoplane (surface of the root), and the endosphere (interior of the root) (16–18). The composition of root microbiomes also shifts throughout the life cycle of rice plants, with individual members displaying reproducible temporal patterns across geographic regions and growing seasons (18, 19). Such highly dynamic spatiotemporal trends emphasize the need to incorporate these sources of variation when exploring the root-associated taxa related to the processes around methanogenesis.

Here, we characterized microbial differences between low- and high-CH_4_-emitting rice cultivars through the growing season by in-depth 16S rRNA sequence analysis of their root microbiomes. Based on previous studies by Simmonds et al. (13), we selected the low-emission hybrid CLXL745 and the high-emission cultivar Sabine, which display divergent CH_4_ emissions late in the season postheading (13). We investigated whether the variation in CH_4_ emissions might be due to either a greater abundance of methanogens, upstream fermenters, and syntrophs in the rhizosphere of Sabine or a greater abundance of methanotrophs in the endosphere or rhizosphere of CLXL745. We also characterized the aerenchyma development in these two cultivars under controlled-growth conditions. We conclude that the cultivars do not differ significantly in aerenchyma or in relative abundance of methanotrophs but that the high-emitting microbiome has an increased relative abundance of methanogenic microbes, as well as compartment-specific consortia of microbes associated with fermentation, sulfate and iron reduction, and acetogenesis.

## RESULTS

The hybrid rice cultivar CLXL745 has been shown to consistently emit less CH_4_ than do other cultivars in a variety of locations and conditions (13, 20–22), whereas Sabine, an inbred cultivar grown in the southeastern rice-producing region of the United States, has been shown to emit significantly more CH_4_ than CLXL745, particularly later in the season (13). In this study, we utilized an experiment in which the two cultivars were grown in an Arkansas field and sampled every 2 weeks over approximately 4 months, constituting the entire life cycle of the plants (18). At each time point, the bulk soil, rhizosphere, rhizoplane, and endosphere were sampled. The rhizosphere and endosphere samples were previously sequenced and analyzed to investigate the dynamics of temporal succession of the microbiome over a growing season (18); however, an in-depth analysis of cultivar variation, particularly in regard to CH_4_ metabolism, had not been performed in that study. Here, we included additionally sequenced samples from the experiment corresponding to the rhizoplane, which represents a critical plant-soil interface, and integrated the previously published raw sequence data from the endosphere and rhizosphere to perform the new analyses detailed below.

### The microbial compositions of the root compartments vary throughout the growing season between Sabine and CLXL745.

Permutational multivariate analysis of variance (PERMANOVA) on Bray-Curtis dissimilarities revealed that compartment, time point, and cultivar were significant main effects and that the interactions between time point and cultivar and between time point and compartment were significant as well (see Table S1A in the supplemental material). To further confirm that the cultivar effect was apparent in each compartment, the data were subsetted by compartment, and PERMANOVA was run on each compartment individually (Table S1B to D). Cultivar and time point were found to be significant in each compartment. To examine if the variation between cultivars in each compartment could be confounded by the location of the plots, PERMANOVA was run on bulk soil samples to check if bulk soils from plots growing CLXL745 varied from bulk soils growing Sabine. The “cultivar effect” (meaning plots growing each cultivar) was not significant, indicating that the variation observed in the compartments is not due to their plots of origin (Table S1E). Principal-coordinate analysis showed the separation of compartments along the first axis, with rhizoplane samples falling between rhizosphere and endosphere samples (Fig. S1A). This observation followed patterns observed in rice microbiome samples in previous studies (16, 18). Furthermore, the rhizoplane samples showed similar temporal dynamics previously elaborated on by Edwards et al. (18). Namely, the microbiota composition of the rhizoplane stabilizes once the plants reach the reproductive stage (Fig. S1C) and have similar temporal shifts in taxa, such as a seasonal increase in Deltaproteobacteria (Fig. S1D). Although these samples were omitted from the previous experiment, these analyses show that the rhizoplane microbiota is not aberrant in its composition or successional patterns.

**FIG 1:**
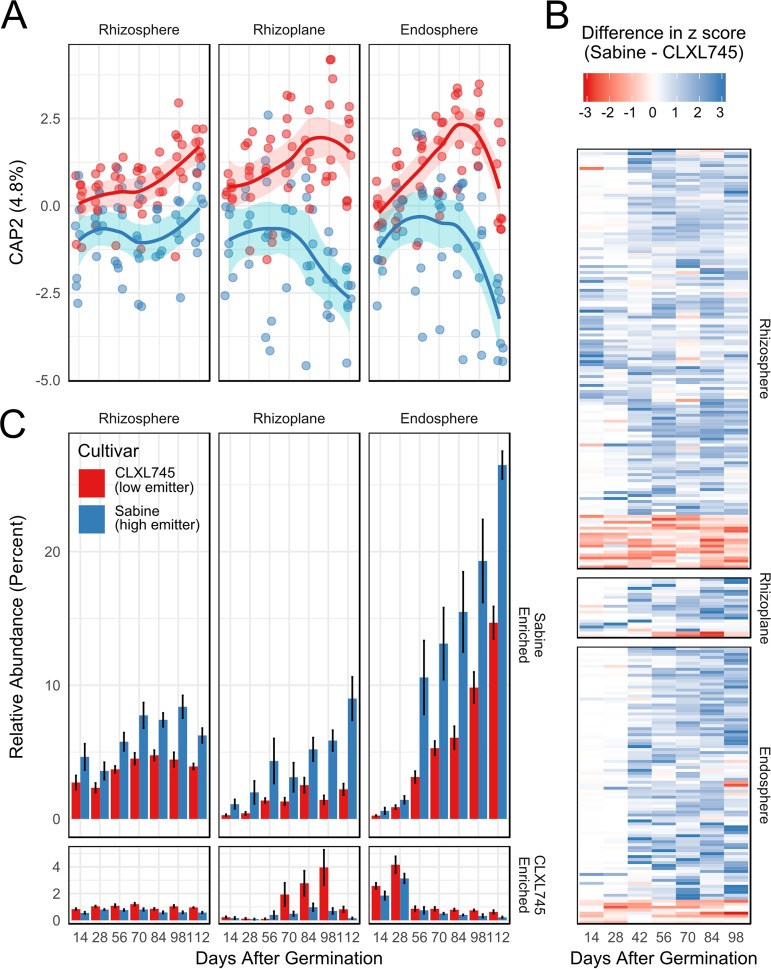
Cultivar significantly shapes the root microbiome. (A) Canonical analysis of principal coordinates controlling for compartment effects. Points are individual samples, whereas the line denotes the cultivar average. The shaded ribbon denotes the standard error (*n* = 6 to 8). The *x* axis represents days after germination and corresponds to the *x* axis in panel C. (B) The difference in Z-scores of each OTU identified as significant (*P* < 0.05) in the likelihood ratio test. Each column represents one time point, and each row is an individual OTU within the compartment denoted on the right. The Z-score is calculated within the OTU across all time points within both cultivars. The difference was calculated by subtracting the Z-score of CLXL745 from Sabine, meaning that positive numbers (blue) are enriched in Sabine over CLXL745, and negative numbers (red) are enriched in CLXL745. White indicates no difference in Z-score. (C) Cumulative relative abundance of Sabine-enriched and CLXL745-enriched OTUs within each compartment. OTUs were defined as Sabine or CLXL745 enriched by averaging the seasonal fold change at each time point between the two cultivars for each OTU in the LRT-derived list. Error bars indicate the standard error (*n* = 6 to 8).

10.1128/mSystems.00897-19.1 FIG S1 Rhizoplane samples follow previously established compartmental and temporal patterns. (A) Principal-coordinate analysis using Bray-Curtis distances. Points are colored by date, whereas shape is determined by compartment. The first two axes are shown. The first axis corresponds to compartment and the second to time. (B) Graphical depiction of CAP described in Fig. 1. The *y* axis is the same as in Fig. 1A. Here, the first axis is shown to correspond with time. (C) Heat map depicting the pairwise similarity between samples of different time points using the Z-score of 1-Bray dissimilarity. The Bray dissimilarity comparisons for the endosphere and rhizosphere samples can be found in Edwards et al. (18). (D) Bar plots of the 10 most abundant taxa in the rhizoplane. Colored dots underneath bars indicate sample age using the same colors as in panels A and B. Download FIG S1, PDF file, 0.1 MB. Copyright © 2020 Liechty et al. 2020 Liechty et al. This content is distributed under the terms of the Creative Commons Attribution 4.0 International license.

10.1128/mSystems.00897-19.5 TABLE S1 (A) PERMANOVA testing the effects of compartment, plot, days, cultivar, days by cultivar, compartment by days, and compartment by cultivar on Bray-Curtis distances. Bulk soil samples were removed prior to analysis. (B to D) PERMANOVA testing the effects of lane, plot, days, cultivar, and cultivar by days of interaction with the rhizosphere (B), rhizoplane (C), and endosphere (D) Bray-Curtis distances. Plot refers to the plots containing one cultivar or the other; cultivars were grown exclusively in four plots each, and therefore this factor is nested within the cultivar term. Lane refers to the library in which the samples were sequenced. The rhizosphere, endosphere, and bulk soil samples were spread out across 4 libraries, so this term is included in the ANOVA, whereas the rhizoplane samples were all sequenced in the same library. This setup causes compartment to be nested in lane, meaning it cannot be included in Table S1A. (E) PERMANOVA testing the effects of time and cultivar (here meaning plots growing one cultivar or the other) and their interaction on bulk soil Bray-Curtis distances. (F) ANOVA testing the effects of cultivar, time, and their interaction on Bray-Curtis distances that have compartment partialled out in the CAP analysis. Download Table S1, DOCX file, 0.02 MB. Copyright © 2020 Liechty et al. 2020 Liechty et al. This content is distributed under the terms of the Creative Commons Attribution 4.0 International license.

A canonical analysis of principal coordinates (CAP) was used to identify variation between cultivars. Since the relative effect size of compartment is large, this variable was partialled out. The results confirmed the significance of the cultivar effect and cultivar-time interaction identified in the above-mentioned PERMANOVA (Fig. 1A and S1B and Table S1F). The first principal-coordinate axis correlated with time, and the second principal-coordinate axis displayed variation due to cultivar. A continual increase in the divergence between the cultivars was observed, although this effect was much larger in the rhizoplane and endosphere than in the rhizosphere (Fig. 1A).

To identify operational taxonomic units (OTUs) whose relative abundances differ between cultivars, a likelihood ratio test (LRT) was performed. We found 141, 20, and 93 OTUs that significantly contributed to cultivar variation in the rhizosphere, rhizoplane, and endosphere communities, respectively (false-discovery rate [FDR], <0.05) (Fig. 1B and Data Set S1). The majority of these cultivar-sensitive OTUs showed no abundance differences between cultivars until after 28 days postgermination, confirming the patterns observed in the CAP analysis (Fig. 1B). The average seasonal log fold change revealed that most of these cultivar-sensitive OTUs were Sabine enriched (123/141 in the rhizosphere, 18/20 in the rhizoplane, and 85/93 in the endosphere). Looking at their cumulative relative abundances further revealed that the magnitude of difference between cultivars increased throughout the growing season within the Sabine-enriched OTUs (Fig. 1C). The Sabine-enriched OTUs also increased in relative abundance throughout the growing season, indicating that many of these OTUs established themselves later in the growing season. These data show that the difference between cultivars becomes more pronounced later in the season, and this difference is driven largely by Sabine-enriched late colonizers.

10.1128/mSystems.00897-19.8 DATA SET S1 The cultivar-sensitive OTUs in each compartment derived through likelihood ratio tests. The *P* value is adjusted through the Benjamini-Hochberg method. This data set also contains the assignment of cultivar-sensitive OTUs within the rhizosphere to six clusters, as well as the trait assignments of the overrepresented families via FAPROTAX. “NA” in the latter column is indicative that the OTU does not belong to an overrepresented family, whereas “none” indicates the OTU is in an overrepresented family, but has no FAPROTAX-assigned trait. Download Data Set S1, XLSX file, 0.05 MB. Copyright © 2020 Liechty et al. 2020 Liechty et al. This content is distributed under the terms of the Creative Commons Attribution 4.0 International license.

### OTUs involved in methanogenesis contribute to the variation between cultivars in each compartment.

**FIG 2:**
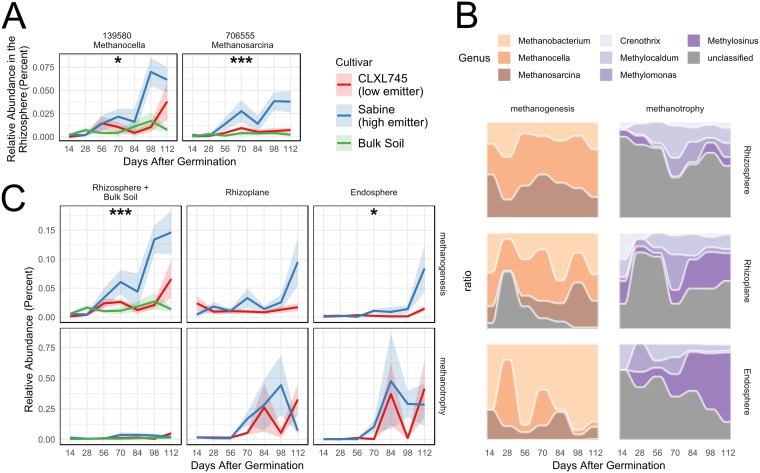
Cultivars vary significantly in methanogen abundances in the rhizosphere but not methanotroph abundance in any compartment. (A) Seasonal trends of OTUs 139580 and 706555, the two methanogens in the list of cultivar-sensitive OTUs detected in the rhizosphere. The colored line indicates the average relative abundance, and the colored ribbon indicates the standard error (*n* = 6 to 8). Asterisks indicate that the OTU was significant in the likelihood ratio test (*, *P* < 0.05; **, *P* < 0.01; ***, *P* < 0.001). Statistical comparisons were only performed between Sabine and CLXL745 samples, and the bulk soil is shown for reference. (B) Total relative abundances of methanogenic archaea and methanotrophic bacteria as defined by FAPROTAX. The shaded colored ribbon indicates the standard error (*n* = 6 to 8). Asterisks indicate that the cultivar term was significant in the ANOVA on variance-stabilized data (*, *P* < 0.05; **, *P* < 0.01; ***, *P* < 0.001). Statistical comparisons were only performed between Sabine and CLXL745 samples. (C) Seasonal shifts in methanogen and methanotroph compositions within each compartment. Color indicates the average relative proportion of methanogens or methanotrophs across both cultivars.

Predictive software, such as the Functional Annotation of Prokaryotic Taxa (FAPROTAX), can be used to identify OTUs in a data set which are likely to display a phylogenetically linked trait of interest. We used FAPTROTAX, which has recently been applied to the rice rhizosphere (23), to identify putative taxa associated with methanogenesis and methanotrophy. FAPROTAX identified methanogenesis-associated OTUs belonging to the genera Methanocella, Methanosarcina, and Methanobacterium. Two methanogenesis-associated OTUs from the genera *Methanocella* and *Methanosarcina* (OTUs 139580 and 706555, respectively) were identified as significant contributors to cultivar variation in the rhizosphere samples (Fig. 2A). Both OTUs had a higher average abundance in Sabine over CLXL745, and this variation was greater later in the season during the growth stages where these cultivars have been shown to be most divergent in CH_4_ emissions (13). No methanotrophic OTUs were significantly differentially abundant between cultivars in any compartment.

The cumulative relative abundances of OTUs associated with methanogenesis and methanotrophy were also compared using analysis of variance (ANOVA) with linear models on data that were variance stabilized using DESeq2 (Fig. 2B and Table S2). Methanogens were significantly enriched in the rhizosphere of Sabine at 98 and 112 days after germination (*P* < 0.05). Methanogen- and methanotroph-associated OTU compositions also changed throughout the season and between compartments (Fig. 2C). *Methanocella* and *Methanosarcina* OTUs were most prominent in the rhizosphere, decreased in abundance in the rhizoplane, and were depleted to an even greater degree in the endosphere. *Methanobacterium* OTUs followed the opposite trend, becoming more prominent from the exterior of the root inward. Similarly, Methylosinus OTUs became the more prominent methanotrophs from the exterior of the root in. In the endosphere, *Methylosinus* OTUs increased in prominence throughout the season as well (Fig. 2C).

10.1128/mSystems.00897-19.6 TABLE S2 ANOVA testing the effect of each trait assigned by FAPROTAX to the overrepresented cultivar-sensitive OTUs within each compartment. ANOVA was run on each trait individually, and then the cultivar term was extracted and corrected for multiple testing using the Benjamini-Hochberg method. Download Table S2, DOCX file, 0.02 MB. Copyright © 2020 Liechty et al. 2020 Liechty et al. This content is distributed under the terms of the Creative Commons Attribution 4.0 International license.

Although differences in methanogen relative abundances were identified between the cultivars in the rhizosphere, it is possible that the relative abundance comparisons between cultivars do not correlate to absolute abundances. For example, one cultivar might support a diverse microbiome with increased microbial load in the rhizosphere compared to another cultivar causing OTUs with relatively lower abundance to have a larger absolute abundance. The absolute abundances of methanogens and methanotrophs are likely to be a better indicator of cultivar effects on CH_4_ emissions. To test if the relative abundances of methanogens and methanotrophs observed correlate with the absolute abundances, we performed quantitative PCR (qPCR) on a methanogen-specific region of the 16S rRNA gene and the alpha subunit of the methane monooxygenase gene (*pmoA*), which is necessary for methanotrophy. This procedure also allowed us to assess whether the absolute abundances of methanogens and methanotrophs varied between cultivars. Since the final two time points showed the greatest differences in cumulative methanogen relative abundances in the rhizospheres of the two cultivars, qPCR was performed on the bulk soil and rhizosphere samples of both cultivars at these time points (Fig. S2A). The bulk soil samples were subsetted by plots growing each cultivar to check if the plots of origin could be affecting the abundances of these markers in our samples; neither marker varied between bulk soils originating from plots growing different cultivars (ANOVA, *P* = 0.3462 for the methanogen-specific 16S rRNA marker and *P* = 0.8469 for the *pmoA* marker), so for further analysis, these samples were not distinguished from each other. The final time point was found to have significant differences between Sabine and both the bulk soil and CLXL745 for the methanogen marker. There was no significant difference between cultivars in *pmoA* abundance (Fig. S2). Furthermore, there was a significant positive correlation between the corresponding methanogen relative abundances from 16S rRNA gene amplicon libraries and absolute abundances from qPCR (*r* = 0.480, *P* = 0.001) but not a significant correlation between methanotroph 16S relative abundance and *pmoA* abundance (*r* = 0.131, *P* = 0.425). This result validated the use of 16S rRNA gene amplicon relative abundances to compare methanogen compositions in this context and confirmed that the high-CH_4_-emitting cultivar had an increased abundance of methanogens over the low emitter. Although there was a weak correlation between the OTUs associated with methanotrophy identified through FAPROTAX and the *pmoA* abundances, both measures confirm that methanotroph abundances do not vary between cultivars.

10.1128/mSystems.00897-19.2 FIG S2 Abundances of methanogen-specific 16S rRNA region and the methanotrophic-associated *pmoA* gene in the bulk soils and rhizospheres of Sabine and CLXL745 for the final two time points in counts per gram of dry weight. Letters above boxplots indicate which pairwise comparisons within that group are significant (*P* < 0.05, Tukey adjustment on log-transformed data). Download FIG S2, PDF file, 0.04 MB. Copyright © 2020 Liechty et al. 2020 Liechty et al. This content is distributed under the terms of the Creative Commons Attribution 4.0 International license.

### Overrepresented families enriched in Sabine are associated with methanogenesis-related processes.

**FIG 3:**
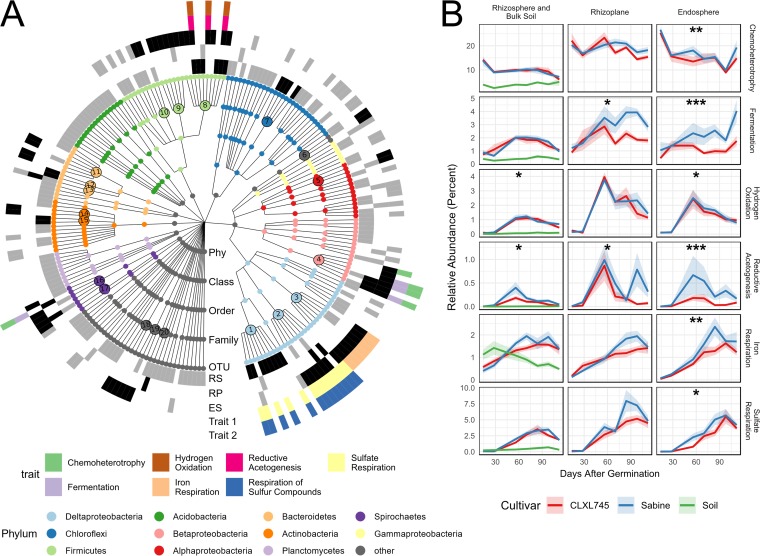
Overrepresented families in the set of cultivar-sensitive OTUs are associated with various anaerobic metabolic traits. (A) Taxonomy dendrogram displaying cultivar-sensitive OTUs in any compartment (*P* < 0.05). The color of each dot represents the phylum (Phy) to which it belongs. A gray or black box in the first three rings indicates that that OTU is cultivar sensitive in the rhizosphere, rhizoplane, or endosphere going from the inside out (RS, rhizosphere; RP, rhizoplane; ES, endosphere). Additionally, a black box means that OTU belongs to a family that is overrepresented among the cultivar-sensitive OTUs in that compartment. The larger numbered circles in the dendrogram are indicative of families that are overrepresented in at least one compartment (hypergeometric test, *P* < 0.05). The corresponding families are found at the end of this text block. The outer two rings indicate traits associated with overrepresented families assigned using FAPROTAX. (B) Relative abundances of all OTUs associated with the traits identified in panel A. The colored shaded ribbon represents the standard error (*n* = 6 to 8). Asterisks indicate that the cultivar term was significant in the ANOVA on variance-stabilized data (*, *P* < 0.05; **, *P* < 0.01; ***, *P* < 0.001). OTUs associated with respiration of sulfur compounds were not included because this list did not vary from the OTUs associated with sulfate respiration. The overrepresented families represented by the numbered circles in panel A correspond to the following families: 1, *Syntrophobacteraceae*; 2, *Desulfovibrionaceae*; 3, *Geobacteraceae*; 4, *Rhodocyclaceae*; 5, unclassified family in the order Ellin329; 6, *Helicobacteraceae*; 7, *Anaerolineaceae*; 8, *Veillonellaceae*; 9, *Ruminococcaceae*; 10, unclassified family in the order *Clostridiales*; 11, unclassified family in the order *Bacteroidales*; 12, BA008; 13, *Bacteroidaceae*; 14, *Cellulomonadaceae*; 15, *Nakamurellaceae*; 16, *Spirochaetaceae*; 17, *Sphaerochaetaceae*; 18, TG3-1; 19, *Ignavibacteriaceae*; and 20, *Phormidiaceae*.

Since there was a significant enrichment of methanogens in the rhizosphere of the high emitter over the low emitter, we hypothesized that other cultivar-sensitive OTUs might be playing a role in the upstream processes related to methanogenesis (i.e., fermentation, syntrophy, etc.). To examine this, hypergeometric tests were performed within each compartment to determine which taxa at each taxonomic rank were overrepresented in the cultivar-sensitive OTUs to identify taxa that are enriched in these lists more than expected by chance (Data Set S2). The methanogenic class *Methanomicrobia* (the class containing methanogenic archaea) was notably overrepresented in the rhizosphere, with two of five *Methanomicrobia* OTUs (OTUs 139580 and 706555 discussed above) present in the list of cultivar-sensitive OTUs (*P* < 0.05). At the family level, the rhizosphere, rhizoplane, and endosphere had five, five, and 14 families overrepresented in the cultivar-sensitive OTUs, respectively (Fig. 3A and Data Set S2). Almost all OTUs belonging to these overrepresented families had greater relative abundance in Sabine than in CLXL745 (Fig. S3).

10.1128/mSystems.00897-19.3 FIG S3 Difference in Z-scores of each OTU overrepresented within the list of cultivar-sensitive OTUs (*P* < 0.05). Each column represents one time point, and each row is an individual OTU within the compartment denoted on the right. The Z-score is calculated within the OTU across all time points within both cultivars. The difference was calculated by subtracting the Z-score of CLXL745 from Sabine, meaning positive numbers (blue) are enriched in Sabine over CLXL745, and negative numbers (red) are enriched in CLXL745. White indicates no difference in Z-score. Download FIG S3, PDF file, 0.06 MB. Copyright © 2020 Liechty et al. 2020 Liechty et al. This content is distributed under the terms of the Creative Commons Attribution 4.0 International license.

10.1128/mSystems.00897-19.9 DATA SET S2 Overrepresented taxa in the cultivar-sensitive list of OTUs within each compartment. Levels of taxa range from phylum to genus. “Level” indicates the taxonomic rank that is being examined. “GeneRatio” indicates the number of OTUs found in the cultivar-sensitive list belonging to that taxon, and the “BgRatio” indicates the number of OTUs found within the relevant compartment that belongs to that taxonomy. RS, rhizosphere; RP, rhizoplane; ES, endosphere. Download Data Set S2, XLSX file, 0.02 MB. Copyright © 2020 Liechty et al. 2020 Liechty et al. This content is distributed under the terms of the Creative Commons Attribution 4.0 International license.

FAPROTAX was again used to identify functions associated with overrepresented families. Only traits assigned to more than one OTU were further considered, but the full list of functional trait assignments can be found in Data Set S1. The overrepresented families mentioned above in all three compartments were associated with sulfate respiration (*Syntrophobacteraceae* in the rhizosphere and *Desulfovibrionaceae* in the rhizoplane and endosphere) (Fig. 3A). Families in both the rhizosphere and the endosphere were associated with reductive acetogenesis and hydrogen oxidation (the genus Sporomusa within *Veillonellaceae* in both the rhizosphere and endosphere) (Fig. 3A). Families in the rhizoplane and endosphere were associated with fermentation and chemoheterotrophy (*Rhodocyclaceae* in both the rhizoplane and endosphere and *Spirochaetaceae* additionally in the rhizoplane) (Fig. 3A). The endosphere additionally contained members of a family associated with iron respiration (*Geobacteraceae*) (Fig. 3A). Although these traits were found to be associated with certain overrepresented families within the cultivar-sensitive OTUs, we wanted to test whether the overall trends of microbes associated with these traits were different between the cultivars in each compartment (Fig. 3B). The data were variance stabilized using DESeq2, and linear models in conjunction with ANOVA were used to identify significant differences. We found taxa associated with reductive acetogenesis, hydrogen oxidation, fermentation, chemoheterotrophy, iron respiration, and sulfur respiration to vary significantly in relative abundance across cultivars and compartments (Table S2). Additional literature was searched to find other functions associated with the overrepresented families of the rhizosphere (Table 1).

**TABLE 1:** Fermentative functions associated with overrepresented families in the list of cultivar-sensitive OTUs^*a*^

Overrepresented taxon	Compartment(s)	Fermentative process (reference)	Source
*Rhodocyclaceae*	RP, ES	Genus *Propionivibrio* (ES, 3/6 OTUs; RP, 2/3 OTUs) ferments sugars, dicarboxylic acids, sugar alcohols, and aspartate to produce propionate and acetate (68)	FAPROTAX
*Spirochaetaceae*	RP	Genus *Spirochaeta* (RP, 1/2 OTUs) produces acetate, ethanol, CO_2_, and H_2_ as fermentative end products (69); previously identified as enriched in endosphere and associated with cellulose degradation (16)	FAPROTAX
*Cellulomonadaceae*	ES	Genus *Actinotalea* (ES, 2/2 OTUs) contains isolates that are cellulose degrading and acetate and formate producing (70)	Literature search
*Veillonellaceae*	RS, RP, ES	Many isolates produce acetate and propionate as fermentative end products (71)	Literature search
*Desulfovibrionaceae*	RP, ES	Produce acetate, CO_2_, and H_2_ through fermentation of lactate and pyruvate (72); *Desulfovibrio* spp. (RP, 2/2 OTUs; ES, 7/7 OTUs) have been characterized to have a syntrophic association with *Methanobacterium* spp., the most abundant methanogens in the endosphere (50)	Literature search
BA008	RS	Produce acetate, propionate, formate, and H_2_ through fermentation (73)	Literature search
*Anaerolineaceae*	RS	“Semisyntrophic,” in that coculture with methanogens significantly stimulated growth (74); produce acetate through fermentation (53)	Literature search
*Syntrophobacteraceae*	RS	Genus *Syntrophobacter* (RS, 5/8 OTUs) act syntrophically with methanogens using H_2_/formate shuttling (75); acetate produced by *Syntrophobacteraceae* consumption of propionate is preferentially consumed by *Methanosarcina* spp. (35)	Literature search

a RS, rhizosphere; RP, rhizoplane; ES, endosphere.

### Clustering analysis identifies OTUs that show seasonal patterns similar to those of methanogen OTUs.

Previous studies have used 16S rRNA gene amplicon data to identify OTUs that cluster with methanogen OTUs in samples in rice paddies or in wetlands that were geographically or compartmentally separated (16, 24, 25). We performed a time series-based clustering using global alignment kernels on the cultivar-sensitive OTUs within the rhizosphere to identify consortia of OTUs that showed similar temporal and cultivar-specific patterns (Fig. S4 and Data Set S1). Methanogenic archaea partitioned to cluster 2, which contained 31 OTUs in total. Eleven of the 31 OTUs in the cluster were of the class *Anaerolineae*, two of which are of the genus Anaerolinea (from the overrepresented family *Anaerolineaceae*), two from the genus Caldilinea, three from the order SBR1031, three from the order GCA004, and one from the order WCHB1-50.

10.1128/mSystems.00897-19.4 FIG S4 Clustering analysis of the cultivar-sensitive rhizosphere OTUs. (A) The trend in Z-scores of OTUs within each cluster. The thick lines are the average trend of the cluster, and the faint lines are the seasonal trend of each individual OTU. Both methanogens are contained in cluster 2. (B) Composition of each cluster, with color indicating the phylum to which the OTUs in each cluster belong. Download FIG S4, PDF file, 0.05 MB. Copyright © 2020 Liechty et al. 2020 Liechty et al. This content is distributed under the terms of the Creative Commons Attribution 4.0 International license.

### Sabine-enriched OTUs generally show an enrichment in the rhizosphere compared to bulk soil samples.

**FIG 4:**
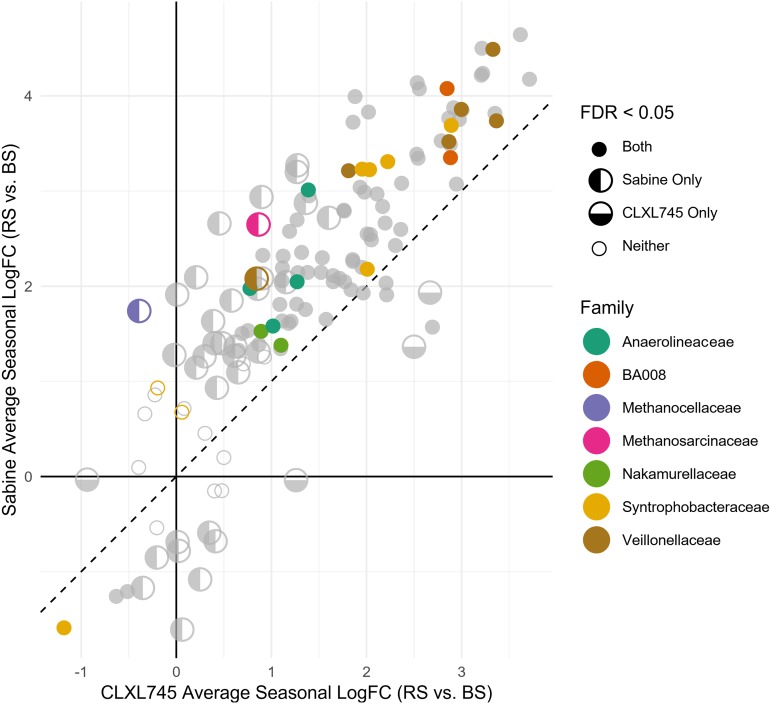
Cultivar-sensitive OTUs are enriched or depleted in a greater degree in the rhizosphere of Sabine than the rhizosphere of CLXL745 when compared to bulk soils (BS) from their respective plots. Each circle represents one OTU that significantly differs between cultivars in the rhizosphere. Colored dots represent the five families that are overrepresented in the list of cultivar-sensitive OTUs in the rhizosphere compared to the total community (hypergeometric test, *P* < 0.05), and the two methanogenic families are represented in the same list. A full circle indicates that that OTU is significantly depleted or enriched in the rhizospheres of both cultivars compared to bulk soil. A half circle filled on the left indicates significant enrichment or depletion in the rhizosphere of Sabine compared to bulk soil but not CLXL745. A half circle filled on the bottom indicates the opposite. An empty circle indicates that neither cultivar is significantly enriched or depleted compared to bulk soil. FC, fold change.

Although rhizosphere OTUs can be classified as enriched in either CLXL745 (low emitter) or Sabine (high emitter), the question remains whether the enrichment of these OTUs in one cultivar is due to an increase in abundance in that cultivar compared to bulk soil or to a depletion in the other cultivar compared to bulk soil. To examine this question, the cultivar-sensitive OTUs on our list were compared between the rhizosphere samples of each cultivar and the bulk soil samples originating from corresponding plots (i.e., rhizospheres from CLXL745 plots compared to bulk soils from CLXL745 plots). The majority of these OTUs had a greater abundance in the rhizosphere of both cultivars than in bulk soil, and the majority of these rhizosphere-enriched OTUs were also enriched in the rhizosphere of Sabine over CLXL745 (Fig. 4). Conversely, OTUs that are depleted in rhizospheres are less abundant in the rhizosphere of Sabine than in that of CLXL745 (Fig. 4). This indicates that Sabine had a larger influence over both those OTUs that are enriched and those OTUs that are depleted. Both methanogen OTUs showed significant enrichment in the rhizosphere of Sabine compared to bulk soil, whereas the methanogens in the rhizosphere of CLXL745 compared to the bulk soil were not significantly different (Fig. 4). All overrepresented families in the rhizosphere LRT-derived list discussed above were also enriched in the rhizosphere over the bulk soil. The *Syntrophobacteraceae* followed a bimodal distribution, with OTUs 620224, 591709, and New.ReferenceOTU1528 showing much less enrichment in the rhizosphere than in the bulk soil.

### Root airspace measurements of cultivars display a complex relationship with microbial taxa distribution and CH_4_ emissions.

An unexpected result of the trait-based analysis described above was an enrichment of microbes associated with fermentation in the endosphere of the high-emitting Sabine over the low-emitting CLXL745. Since the endosphere is a relatively aerobic environment, and the fermentation processes that support methanogens are anaerobic, we hypothesized that the observed variation might be due to either structural variation of the root between cultivars allowing for greater activity of anaerobic metabolism or to an increased substrate availability in the anaerobic or microaerobic sections of the root. In support of the first hypothesis, Jiang et al. found that in a comparison of two cultivars, a higher-performing cultivar had a greater airspace than did a lower-yielding cultivar, which could account for increased oxygen diffusion into the root and potentially an increase in methanotrophy (14).

**FIG 5:**
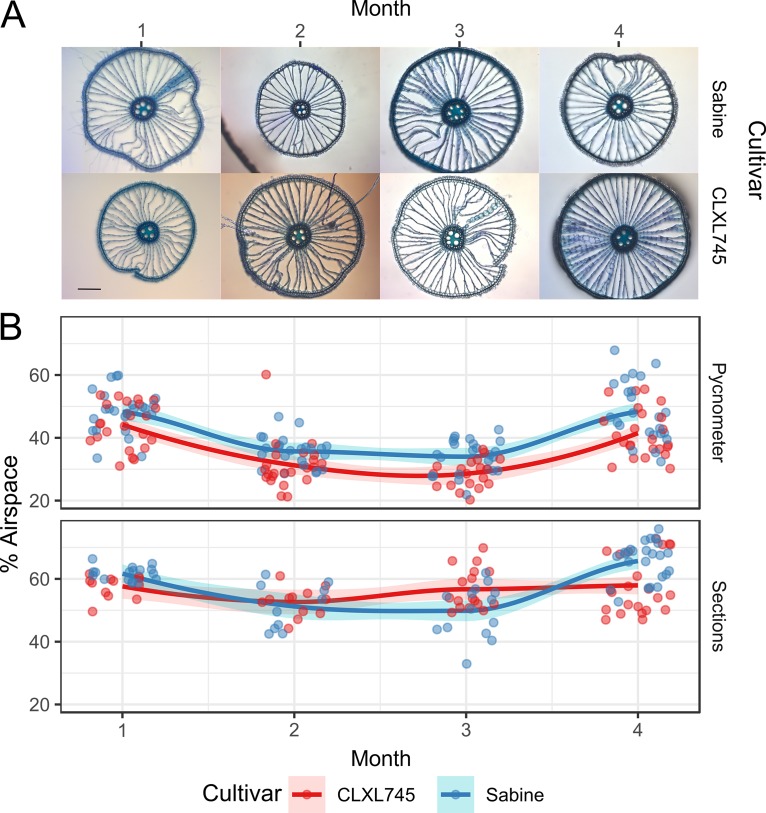
Airspaces of Sabine and CLXL745 over time. (A) Cross-sections indicative of those used to quantify the airspace in panel B. All images are at the same magnification. Black scale bar = 100 μm. (B) Percent airspace calculated using pycnometers (top) and area of sections (bottom). Dots in the top graph represent individual pycnometer measurements (*n* = 3 per plant). Dots in the bottom graph represent measurements from individual cross-sections of roots (*n* = 3 to 5 per root). Lines are the average of each sample (determined by averaging the subsamples). The shaded ribbons indicates the standard error (*n* = 4).

We therefore investigated whether Sabine had a reduced airspace compared to CLXL745, resulting in more anaerobic/microaerobic environments where fermentation can occur. To test this, we measured aerenchyma in both cultivars during four monthly time points throughout the life cycle in a greenhouse experiment. The proportion of root space occupied by the aerenchyma was measured by two methods (26). The first method was direct observation of cross-sections of similar-sized mature roots of the two cultivars (Fig. 5A). The second method was indirect measurement, using the pycnometer method, which measures total airspace volumes in a selection of roots; the volumes were used to compute the proportional airspace in that selection of roots (Fig. 5B).

We used ANOVA and linear modeling to determine significant factors affecting aerenchyma variation using both methods (Table S3). Conflicting results were obtained, which are likely due to the differences in measurement types, wherein the cross-sections and the pycnometer measure the proportional air capacities of individual mature roots and of the total roots, respectively. For example, the pycnometer measurements can be influenced by factors such as increased tillering, which produces a greater fraction of younger roots with undeveloped aerenchyma. The cross-section measurements indicated that there were no significant differences in aerenchyma sizes between the cultivars, i.e., roots of similar diameter did not differ in aerenchyma area in the cross-sections. We did observe a significant difference in the volume percentages between the cultivars in the pycnometer measurements (Table S3). However, using this assay, the high-emission cultivar Sabine showed a proportionately greater aerenchyma volume than did CLXL745, which is the opposite of the result expected, both from our hypothesis and from the predictions of Jiang et al. (14).

10.1128/mSystems.00897-19.7 TABLE S3 (A) ANOVA testing the effects of time, cultivar, and their interaction on section measurements. (B) ANOVA testing the effects of time, cultivar, and their interaction on pycnometer measurements. (C) Contrasts between CLXL745 and Sabine at each of the four time points. Download Table S3, DOCX file, 0.01 MB. Copyright © 2020 Liechty et al. 2020 Liechty et al. This content is distributed under the terms of the Creative Commons Attribution 4.0 International license.

## DISCUSSION

### Microbial variation between cultivars suggests an increased relative abundance of methanogens in the high-CH_4_-emitting Sabine.

In this study, we investigated the possible factors underlying differences in CH_4_ emissions between the high-emission rice cultivar Sabine and the low-emission rice variety CLXL745. We identified two methanogen OTUs belonging to the genera *Methanocella* and *Methanosarcina* that were enriched in the rhizosphere of Sabine compared to the rhizosphere of CLXL745. Both of these OTUs showed greater variation in relative abundance during the end of the season, which correlated with increased variation in CH_4_ emissions posttransition to the reproductive stage (Fig. 2) (13). Total methanogen relative abundance was shown to be significantly enriched in the Sabine rhizosphere over the CLXL745 rhizosphere during the final two time points. This divergence notably correlates with the seasonal divergence in CH_4_ emissions, which is most prominent later in the season (13). These findings were confirmed by qPCR, validating the conclusions drawn from the analysis of the relative abundances of the 16S rRNA gene sequences. Furthermore, it was demonstrated that these two methanogenic OTUs were significantly increased in the rhizosphere of Sabine over bulk soil, whereas CLXL745 did not vary from bulk soil (Fig. 4). This further supports the hypothesis that the methanogens are truly enriched in the rhizosphere of the high emitter and not depleted in the rhizosphere of the low emitter.

*Methanocella* spp., hydrogenotrophic methanogens formerly known as Rice Cluster I have been shown to incorporate more plant-derived carbon than do other methanogenic groups (27). The inclusion of this taxon among the enriched methanogens could be indicative of differences in the exudation of plant carbon sources to be a large contributor to the differences in methanogen abundances. *Methanosarcina* spp. are able to utilize all three methanogenic pathways (utilization of H_2_ and CO_2_, methylated compounds, and acetate) (4). *Methanosarcina* spp. have a low affinity for acetate but outcompete the strictly acetoclastic methanogens Methanosaeta spp. at higher temperatures, typical of those occurring during the growing season in this study (28, 29). *Methanosarcina* spp. are also thought to dominate over *Methanosaeta* spp. at higher acetate concentrations, which could be the case in the organic carbon-rich rhizosphere (30). *Methanosarcina* spp. can oxidize acetate, producing the necessary components for hydrogenotrophic methanogenesis (31). Thus, it is reasonable that in this study, especially in the absence of strictly acetoclastic methanogens (Fig. 2C), that the increased abundance of *Methanosarcina* OTUs could utilize hydrogen and CO_2_ or acetate to produce CH_4_, or it could enable the oxidization of acetate to further promote hydrogenotrophic methanogenesis by *Methanocella* spp.

Contrary to other studies investigating methanotrophs in rice hybrids, we observed no variation between cultivars in methanotroph relative abundances using 16S rRNA sequencing or in absolute abundances using qPCR on the *pmoA* gene. Furthermore, the cultivars did not vary in methanotrophic syntrophs in the way they varied in methanogenic syntrophs. For example, laboratory isolation or enrichment of methanotrophs is often accompanied by species in the genus Hyphomicrobium (32). *Hyphomicrobium* spp. can remove methanol, an inhibitor to methanotrophic growth. Although present in our data set, no *Hyphomicrobium* OTUs were present in the cultivar-sensitive lists. Although methanotrophs abundances did not vary across cultivars, they did vary across compartments. Notably, type I methanotrophs (Methylocaldum, *Methylosinus*, and Crenothrix spp.) were in greater relative abundance in the rhizosphere, whereas type II methanotrophs (*Methylosinus* spp.) were more abundant in the endosphere. It has been shown that high concentrations of CH_4_ in soil stimulate type I but not type II methanotrophs, which supports our results (33). In addition, *Methylosinus* spp. may be enriched within the endosphere due to their ability to utilize methanol, which is produced by demethylation of pectin in the cell walls of plants (34).

Although the qPCR results comparing the relative abundances of methanogens and absolute abundances of the methanogen-specific 16S rRNA correlated significantly, the results for methanotrophs were less clear due to the weak correlation between 16S rRNA relative abundance and *pmoA* absolute abundance. A previous study indicated that the community composition of methanotrophs varies drastically when sequenced with methanotroph-specific 16S rRNA genes or *pmoA* genes, which could contribute to the variation seen here (33). It is also possible that the assignment of OTUs associated with methanotrophy with FAPROTAX missed previously uncharacterized methanotrophs. Furthermore, FAPROTAX can only identify associations if OTUs are classified at the family or genus level, so methanotrophic OTUs not classified at these levels would be missed. This demonstrates some of the limitations of assigning traits based on 16S rRNA gene amplicon data and should be taken into consideration when considering the other trait associations discussed in this study.

### Trait-based analysis suggests an increase in anaerobic microbial metabolism across all compartments in Sabine, leading to better conditions for methanogenesis.

Since methanogen OTUs are enriched in the rhizosphere of the high emitter, we were able to identify patterns of microbial succession associated with processes upstream of methanogenesis. Two factors could lead to an increased abundance of methanogenesis, the availability of precursor substrates, and a highly reduced environment. Anaerobic metabolism involving iron and sulfate is more energetically favorable than methanogenesis, meaning that these electron acceptors must be depleted before methanogenesis can occur (9, 11). For example, it has been demonstrated in rice paddies that the addition of sulfate can reduce CH_4_ emissions by 70% (35). Our study found overrepresented families associated with both sulfate reduction (*Syntrophobacteraceae* in the rhizosphere and *Desulfovibrionaceae* in the rhizoplane and endosphere) and iron reduction (*Geobacteraceae* in the endosphere). The overall seasonal trends of taxa were associated with iron reduction peaking earlier in the season (70 days postgermination), followed by a peak in sulfate reduction (98 days postgermination) and a continued increase in the relative abundances of methanogens throughout the growing season (Fig. 2B and 3B). This follows the theoretical progression of electron acceptor usage, since the reduction of iron is more favorable than the reduction of sulfate, which is more favorable than methanogenesis. An increase in these activities earlier in the season (as suggested by the association of overrepresented families with these traits) could lead to more favorably reduced conditions earlier on in the season for methanogenesis to occur.

**FIG 6:**
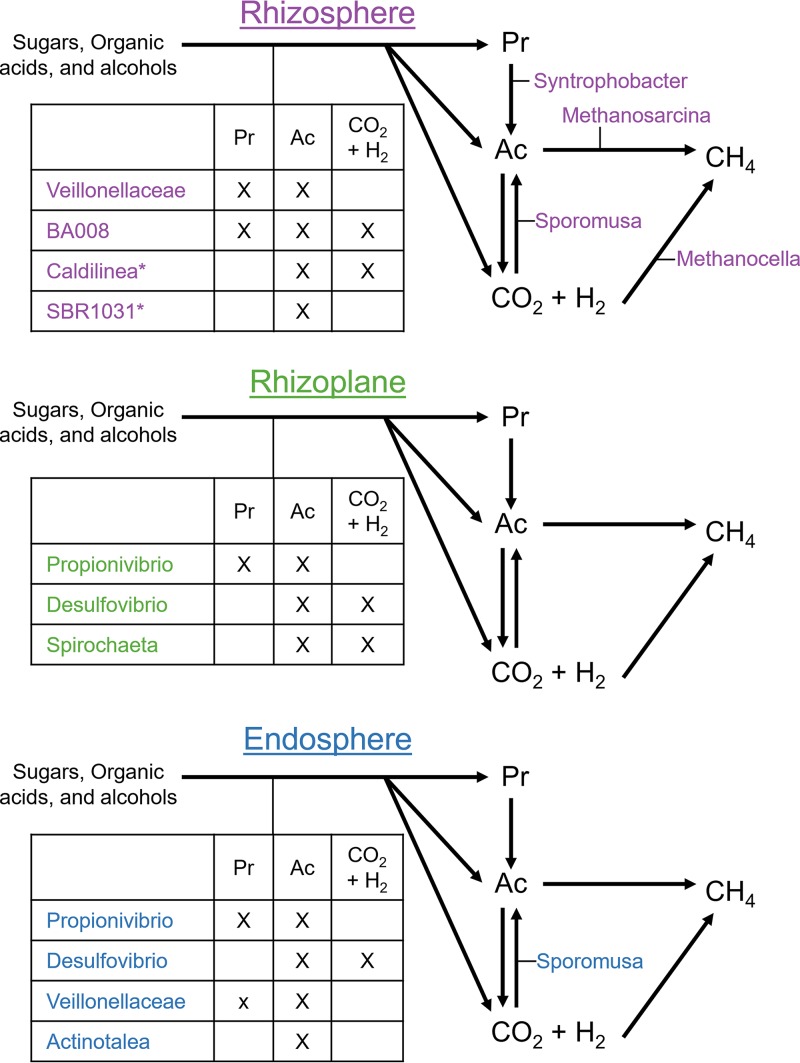
A summary of potential metabolic roles carried out by microbes enriched in Sabine (high emitter) over CLXL745 (low emitter). The table shows fermentative end products of taxa listed on the left, as discussed in the text. All microbes listed are from enriched families, except those indicated by an asterisk, which are taxa that clustered with methanogens in the rhizosphere. Pr, propionate; Ac, acetate.

An increased methanogen relative abundance could also be stimulated by an increased substrate availability. Some microbes can ferment carbon inputs to a variety of carbon sources, including organic acids, alcohols, propionate, acetate, H_2_, and CO_2_, the last three of which can be used as the substrates for methanogenesis. The production of acetate and propionate is particularly notable, because 70% and 23% of emitted CH_4_ goes through acetate and propionate as intermediates, respectively (35, 36). A study comparing high- and low-CH_4_-emitting cultivars has found a greater abundance of acetate in the rhizosphere of the high emitter, further confirming the importance of acetate as an important intermediate (37). Our study identified several taxa associated with fermentation, as summarized in Table 1 and Fig. 6. Furthermore, the seasonal trends of the cumulative OTUs associated with fermentation are significantly greater in the endosphere and rhizoplane of Sabine than in those of CLXL745. This increased abundance of fermentation-associated OTUs could be indicative of a greater abundance of methanogenic precursor molecules which could stimulate methanogenesis.

We hypothesized that this increase in fermentation-associated OTUs was due to a greater microaerobic/anaerobic sections of the Sabine root due to a less-developed root airspace; however, we found no significant difference in the proportion of root cross-sections occupied by aerenchyma and a significantly greater proportion of aerenchyma volume in Sabine. This result differs from our expectations, as well as from the model of Jiang et al. (14), which would predict that the higher-yielding/lower-emission cultivar CLXL745 will have proportionally greater aerenchyma space than the lower-yielding/higher-emission cultivar Sabine. Our data further diverge from the results of Jiang et al. (14) in changes in methanogens and methanotroph abundances, in that we found an increase in methanogen abundance in our high-emitting cultivar, whereas they found an increase in methanotroph abundance in their low-emitting cultivar. We conclude that the genetic factors involved in genotype-dependent fermentative OTU abundance in our study are unlikely to act by a simple mechanism involving control of root porosity. In our study, we focused on root airspace due to recent reports that variation in airspace between hybrids and other rice cultivars influence CH_4_ emission (14). In addition, other morphological and physiological traits have also been correlated with CH_4_ emissions, including above- and below-ground biomass (38), root exudation rate (39), and variation in the root-shoot transition zone (40). These traits could be affecting the composition of methanogenic, methanotrophic, or other related taxa and are physiological factors that could be further studied between these cultivars.

Another interesting trait associated with some overrepresented families of both the rhizosphere and endosphere was reductive acetogenesis (Fig. 3). Acetogens use the Wood-Ljungdahl pathway to produce acetate from CO_2_ and H_2_ (41). This would put them in competition with hydrogenotrophic methanogens, such as *Methanocella* spp., due to utilization of the same substrates; however, methanogenesis is more energetically favorable than is acetogenesis, meaning that acetogens would be outcompeted and must resort to other modes of metabolism (41). Interestingly, microbes that act acetogenically in culture will oxidize acetate, running the Wood-Ljungdahl pathway in reverse, when in the presence of a syntroph (41, 42). In both the rhizosphere and the endosphere, *Sporomusa* is an overrepresented genus associated with acetogenesis. This genus has previously been observed in experiments studying the incorporation of CO_2_ into acetate on rice roots (43). However, when *Sporomusa* spp. are grown in the presence of Desulfovibrio spp., which is also an overrepresented genus in the endosphere, no acetate is formed, and methanol is oxidized to CO_2_ and H_2_ (42). This is indicative that the *Sporomusa* spp. in these samples might be performing activities other than acetogenesis which could further promote methanogenesis. It is noteworthy that taxa associated with acetogenesis peak in the middle of the season across all compartments, which does not follow the trend of methanogens during that time period, with whom they theoretically could be competing for substrates. Many acetogens have high metabolic flexibility and are additionally able to ferment, which could cause this initial peak (44).

It is surprising that taxa associated with methanogenesis, fermentation, and acetogenesis are enriched in the aerobic endosphere of one cultivar over the other, considering that these are anaerobic processes. This is also not the first time we have observed strictly anaerobic taxa in the endosphere compartment; Edwards et al. (16) identified an enrichment of methanogenic *Methanobacterium* OTUs in the endosphere, which we again see in this study (Fig. 2). Furthermore, a study has recently shown that *Methanobacterium* OTUs were more enriched in the endosphere of rice plants than in other native plant species growing in the same field, indicating that *Methanobacterium* spp. have a unique interaction with rice (45). Previous studies have correlated the activity of superoxide dismutase with oxygen tolerance in some taxa, including *Methanobacterium,* as well as some anaerobic taxa discussed above (e.g., *Desulfovibrio* and Propionivibrio) (46–48). However, *Methanosarcina* OTUs, which have also been shown to have a tolerance to oxygen via superoxide dismutase (49), are enriched in the rhizosphere but not the endosphere, indicating that the above-mentioned taxa are able to persist in the endosphere due to other unknown factors.

To summarize, multiple enriched families in all three compartments of Sabine over CLXL745 have been associated with fermentation and the production of propionate, acetate, CO_2_, and H_2_. Interestingly, some of the enriched taxa have been previously found to be associated with methanogenic archaea. The rhizosphere of Sabine is enriched with *Syntrophobacter* OTUs, isolates of which have been shown to degrade propionate to acetate and have been closely associated with *Methanosarcina* spp. (35), of which one OTU is also enriched in the rhizosphere (35). The Sabine endosphere is enriched for *Desulfovibrio* OTUs, which is associated both with the acetogens of *Sporomusa* spp. as well as the dominant endosphere methanogens, *Methanobacterium* spp. (42, 50). This demonstrates the potential for unique consortia in each compartment contributing to an increase in abundance of methanogenic substrates for the corresponding archaea.

### Clustering analysis reveals a potential syntrophic relationship between the class *Anaerolineae* and methanogens.

In addition to OTUs that are generally overrepresented in one cultivar over the other, clustering analysis allows us to identify OTUs that potentially interact more directly with methanogens. Previous studies have identified OTUs that cluster with methanogens that are spatially separated; this allows for the identification of OTUs related to methanogens across a much larger diversity of environments, including across diverse plant compartments and geographic locations (16, 24, 25). Clustering across a season will identify OTUs more specifically linked to methanogen metabolism as substrate availability and soil redox potential change over time. Some of the taxonomies of methanogen-clustering OTUs have previously been identified as methanogen clustering or CH_4_ production clustering in other experiments over a variety of conditions, including the phylum *Planctomycetes* , order iii1-15, Geobacter, Sphingomonas, family *Ignavibacteriaceae*, class *Phycisphaerae*, and *Anaerolineae* families *Anaerolineaceae*, A4b, and SHA-31 (16, 24, 25).

However, these studies did not show significant positive correlations or clustering with other taxa identified in this study, including multiple families of the class *Anaerolineae*. The *Anaerolineae* family *Caldilinea* isolates have been shown to produce acetate, CO_2_, and H_2_ through fermentation (51, 52). Genome sequences from the uncultured SBR1031 have been shown to contain key genes in pathways necessary for acetate production through fermentation (53). These results show that temporal clustering identifies key taxa that cooccur with methanogens and could produce fermentation products that were not identified in previous spatial clustering analyses. Specifically, the presence of 11 OTUs in the class *Anaerolineae* out of a total 30 OTUs in the cluster suggest that this class could have a syntrophic relationship with methanogens.

In conclusion, this study utilized a high-emission cultivar and a low-emission cultivar to investigate the relationships between emission differences and the abundances of CH_4_-cycling microbes in their root-associated microbiomes. The high-CH_4_-emitting cultivar, Sabine, had an increased relative abundance of methanogens, as well as taxa associated with upstream processes related to methanogenesis (fermentation, acetogenesis, and iron and sulfate reduction) but no significant differences in methanotrophs relative to the low emitter CLXL745. The enrichment of fermentative microbes in the endosphere of the high emitter does not arise from reduced airspace in the roots, suggesting that the cultivars vary in the abundances of fermentation-associated taxa due to increased substrate availability in the exudates from the roots of the high emitter. The identity of these upstream taxa and the factors that control their abundance could provide avenues for efforts to manipulate plant influence over the microbiome to reduce CH_4_ emissions in rice.

## MATERIALS AND METHODS

Compartment separation, sample processing, and sequence processing have recently been published in *Bio-Protocols*, and a more in-depth explanation of the 16S rRNA gene amplicon pipeline can be found there (54).

### Arkansas field experiment sampling and DNA extraction.

Samples were grown in 8 different plots with 4 plots per cultivar. Two individual plants were collected from each plot at each time point and treated as individual replicates for a total of 8 replicates per factor combination. Bulk soils were also sampled from the same 8 plots. The rhizosphere and endosphere data used in this paper were previously published by Edwards et al. (54). The rhizoplane samples were not included in that study, though the samples were collected at the same time as the endosphere and rhizosphere samples and frozen at −80°C. These samples were not included in the original study because the authors were unsure if the samples would be compromised in transport and were not necessary for the temporal dynamics explored in that paper. Due to the significant insight rhizoplane samples could add to the taxa involved in methane dynamics, the rhizoplane samples were sequenced to check for quality to include in further analysis. Rhizoplane samples corresponding to 42 days after germination were compromised before library preparation, so all samples corresponding to that time point were removed in downstream analyses. Further information about the field setup and sample collection can be found in the paper by Edwards et al. (54). Rhizoplane samples were thawed at room temperature, and extractions were performed using the Mo Bio PowerSoil DNA isolation kits.

### 16S rRNA gene amplicon library preparation.

Libraries were prepared using dual-index primers, as previously described (16, 18, 55). PCR was performed using the Qiagen HotStar HiFidelity polymerase kit. Touchdown PCR was used to amplify the samples with the following steps: 95°C for 5 min, 35 cycles of 95°C for 45 s, 50°C for 1 min, and 72°C for 1 min, and 72°C for 10 min. A negative control was included for each sample to identify contamination, which was identified using a 1% agarose gel. AMPure beads were used to remove the primer dimer, and the Qubit high-sensitivity (HS) assay kit was used to quantify the concentrations. Samples were pooled, gel purified, and sequenced using the Illumina MiSeq machine on a 2 × 250 paired-end run.

### Sequence processing.

The rhizoplane paired-end reads were combined with the rhizosphere and endosphere paired-end reads and demultiplexed with custom scripts (https://github.com/RiceMicrobiome/Edwards-et-al.-2014/tree/master/sequencing_scripts). PANDAseq was used to align the endosphere, rhizosphere, and rhizoplane reads (56). Sequences with ambiguous bases and reads over 275 bp were discarded. OTUs were clustered at 97% using UCLUST (57). An open-reference strategy was used against the 13_8 Greengenes 16S rRNA sequence database (58). OTUs with a name beginning with “New.ReferenceOTU” or “New.CleanUp.ReferenceOTU” were generated during the *de novo* clustering stage of the open-reference algorithm. Chloroplast and mitochondrial OTUs were then removed, and OTUs occurring in less than 5% of the samples were removed as well. Sequencing depths varied from 3,985 to 161,535 reads, with a median of 37,239 reads. OTUs were divided by the sequencing depth and multiplied by 1,000 to form relative abundances in units of per mille for analysis. However, all plot relative abundances are shown in percentages. Some samples had large spikes of *Gammaproteobacteria* in all compartments; however, the spikes did not correlate across compartments so were likely introduced through contamination. These spikes were largely made up of a single OTU, 839235 of the family *Aeromonadaceae*, which has been found in much lower abundances in another data set, which averaged 0.017% across all samples (17). Therefore, samples that had a relative abundance of *Gammaproteobacteria* two standard deviations greater than the mean were removed. No more than 2 samples were removed from any factor combination, meaning that the replicates per factor combination ranged from 6 to 8 samples.

### qPCR quantification.

The weights of the original rhizosphere samples were not recorded during the original sampling, so the remaining frozen rhizosphere samples were thawed and reextracted using the Mo Bio PowerSoil DNA isolation kit. The protocol was followed as normal, but the initial weight was recorded before the extractions were performed. The thawed rhizosphere samples were briefly dried in an oven, and approximately 100 mg (dry weight) was extracted. Three samples did not have enough remaining sample to be extracted (<50 mg) and were excluded from the extraction. The qPCR method was derived from previously published methods for methanogen quantification using methanogen-specific 16S rRNA primers (12). The samples were diluted 1/10 to reduce the effects of PCR inhibitors. Previously published methanogen-specific primers were used (MET630F, GGATTAGATACCCSGGTAGT; MET803R, GTTGARTCCAATTAAACCG) (12). A PCR-cloned 16S rRNA gene fragment extracted from environmental samples was used as a standard. Triplicates of each sample were run, and replicates that disagreed with the other two replicates were excluded. The gene copy number of each sample was calculated using the values from the serially diluted standard. Those copy numbers were corrected to reflect the DNA copy number per gram of dried soil. The qPCRs were prepared with Bio-Rad iTaq Universal SYBR green Supermix, and the qPCR program for methanogen-specific 16S rRNA region was that reported by Su et al. (12), as follows: 95°C for 7 min, followed by 54 cycles of 40 s at 95°C, 1 min at 60°C, and 40 s at 72°C. The melting curve was from 65°C to 95°C, increasing at 0.5°C increments for 5 s each. The qPCR primers a189 and mb661 were used to amplify the *pmoA* gene, with thermocycler settings of 94°C for 4 min and 35 cycles of 94°C for 30 s, 56°C for 30 s, and 72°C for 1 min, followed by the same melt curve described above.

### Statistical analysis.

All statistical analyses were carried out in R version 3.5.1 (59). PERMANOVA was performed using the adonis() function, Bray-Curtis dissimilarities were calculated with the vegdist() function, and canonical analyses of principal coordinates were performed with the capscale() function from the vegan package (60). Unconstrained principal-coordinate analysis was performed using the pcoa() function in the ape package (61). Likelihood ratio tests and differential abundance analyses were performed using DESeq2 (62). The models used in the likelihood ratio test were the full model, sequencing lane + time point + cultivar, compared to a reduced model, sequencing lane + time point. These models were run on data subsetted by compartment. Hypergeometric tests were performed by taking the list of taxonomies at each taxonomic level from the list of cultivar-sensitive OTUs and comparing them to the list of the same taxonomic rank of all OTUs present within each compartment. Hypergeometric tests were performed with the enricher() function from the package clusterProfiler using default parameters with no upper or lower size cutoff (63). Variance stabilization was performed with the vst() function from DESeq2, which normalizes the variance within each OTU while accounting for library size (62). Clustering was performed on Euclidean distances of Z-score-transformed relative abundances ([value − mean]/standard deviation) using the function hclust from the stats package (59). Clusters were determined using the function tsclust() from the package dtwclust (64). The number of clusters was determined by graphing the mean sum of squares for a number of clusters ranging from 2 to 10 and identifying where the slope leveled out. Linear models and ANOVA were performed using lm() and anova(), respectively, from the stats package (59). qPCR results were analyzed using log-transformed data, and posttransformation normalization was checked using normal Q-Q plots from the stats package. All plots were generated with the ggplot2 package (65). All scripts are posted on GitHub (https://github.com/zliechty/RiceCH4).

### Greenhouse experiment setup.

The aerenchyma measurement experiment was carried out in a UC Davis greenhouse in the summer of 2018 in a randomized complete block design. Four 23-gallon tubs were arranged in a 2 by 2 layout, with each tub holding 16 plants (8 of each cultivar) in 5.5- × 5.5-in. pots. Plants were sampled monthly, beginning 1 month after germination. At each time point, two plants of each cultivar were sampled.

### Pycnometer measurements.

The pycnometer measurements followed an established protocol (66). Soil was washed from the roots using tap water. Once all soil was removed, approximately 1 g of root taken from the first 10 cm below the root-shoot junction was sampled. Three independent replicates per plant were sampled. Samples were patted dry with paper towels, weighed, and cut into approximately 1-cm pieces. This was put into a pycnometer and weighed. The pycnometer was then vacuum infiltrated for 5 rounds of 5-min intervals or until bubbles stopped rising upon vacuum initiation, and then they were weighed again. The equation (P_vr – P_r)/(P_w + R – P_r) was used to calculate airspace, where P_w is the weight of the pycnometer with only water, P_r is the weight of the pycnometer with roots and water, P_vr is the weight of the pycnometer with vacuum-infiltrated roots, and R is the weight of the dry roots. Water was brought to room temperature before beginning measurements. The three replicates per plant were averaged before statistical analysis was performed.

### Cross-section preparation, imaging, and analysis.

Plants were washed in the same fashion as described above in “Pycnometer measurements.” A 3-cm section of root was cut with a razor blade and vacuum infiltrated with FAA (50% ethanol 95%, 5% glacial acetic acid, 10% formalin, 35% water) for 10 min. The root sample was then embedded in 5% agarose and flash frozen with liquid nitrogen. The plug was then vacuum infiltrated with FAA for 10 min and left in FAA overnight. The plugs were then rehydrated in a series of 70%, 50%, 30%, and 10% ethanol washes, each lasting 30 min. The plugs were then stored in water until sectioning. Sectioning was performed with a Leica VT1000 vibratome, with sections ranging from 200 to 300 μm. Root sections were then dyed with 0.1% toluidine blue for 30 s and rinsed with water. Images were taken using Zeiss Axioskoop2 plus microscope with an AxioCam HRc camera. Images were analyzed in ImageJ (67) by dividing the area of airspace by the total area of the root section. Multiple sections of each root were taken, analyzed, and then averaged for statistical analysis.

### Data availability.

The rhizosphere and endosphere reads can be found at the Sequence Read Archive of NCBI under BioProject accession number PRJNA392701. The rhizoplane reads can be found at BioProject accession number PRJNA598892.
