# Supplementary material for: Comparative Analysis of Root Microbiomes of Rice Cultivars with High and Low Methane Emissions Reveals Differences in Abundance of Methanogenic Archaea and Putative Upstream Fermenters
Source: mSystems. 2020 Feb 18;5(1):e00897-19. doi: 10.1128/mSystems.00897-19 (PMC7029222; doi:10.1128/mSystems.00897-19)
Supplement: FIG S1 [file mSystems.00897-19-sf001.pdf]

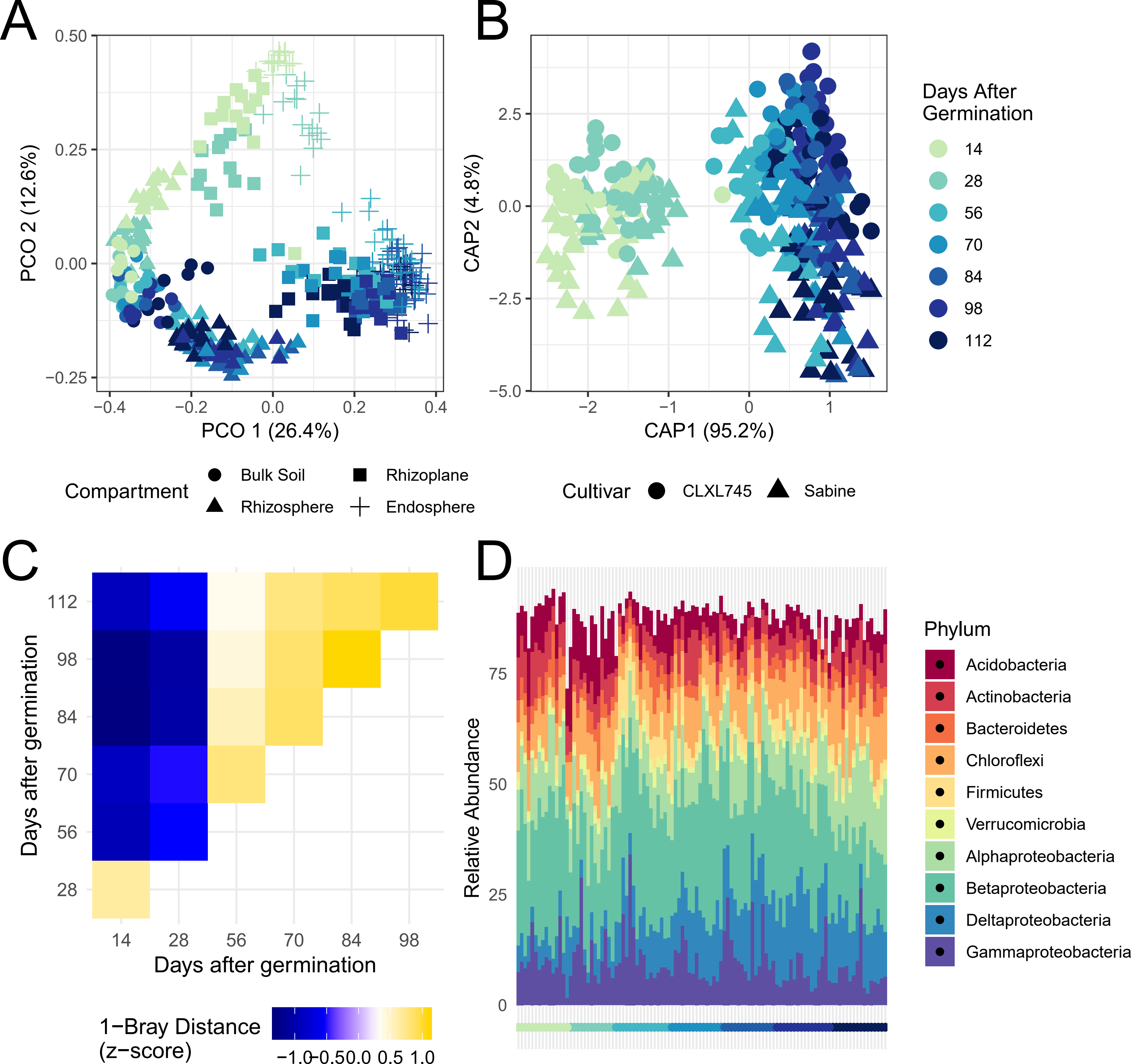

A
B
C
D
PCO 1 (26.4%)
PCO 2 (12.6%)
CAP1 (95.2%)
CAP2 (4.8%)
Days After Germination
14
28
56
70
84
98
112
Compartment
Bulk Soil
Rhizoplane
Rhizosphere
Endosphere
Cultivar
CLXL745
Sabine
Days after germination
1−Bray Distance (z−score)
Relative Abundance
Phylum
Acidobacteria
Actinobacteria
Bacteroidetes
Chloroflexi
Firmicutes
Verrucomicrobia
Alphaproteobacteria
Betaproteobacteria
Deltaproteobacteria
Gammaproteobacteria
