# Supplementary material for: Comparative Analysis of Root Microbiomes of Rice Cultivars with High and Low Methane Emissions Reveals Differences in Abundance of Methanogenic Archaea and Putative Upstream Fermenters
Source: mSystems. 2020 Feb 18;5(1):e00897-19. doi: 10.1128/mSystems.00897-19 (PMC7029222; doi:10.1128/mSystems.00897-19)
Supplement: TABLE S1 [file mSystems.00897-19-st001.docx]

**A**

|  | Df | SumsOfSqs | MeanSqs | F.Model | R2 | Pr(>F) |  |
| --- | --- | --- | --- | --- | --- | --- | --- |
| Compartment | 2 | 15.142 | 7.5709 | 57.684 | 0.21931 | 0.001 | *** |
| Plot | 1 | 0.38 | 0.3798 | 2.894 | 0.0055 | 0.004 | ** |
| Days | 1 | 9.687 | 9.6871 | 73.808 | 0.14031 | 0.001 | *** |
| Cultivar | 1 | 0.474 | 0.4745 | 3.615 | 0.00687 | 0.003 | ** |
| Days:Cultivar | 1 | 0.303 | 0.3029 | 2.308 | 0.00439 | 0.018 | * |
| Compartment:Days | 2 | 1.977 | 0.9887 | 7.533 | 0.02864 | 0.001 | *** |
| Compartment:Cultivar | 2 | 0.26 | 0.1298 | 0.989 | 0.00376 | 0.417 |  |
| Residuals | 311 | 40.818 | 0.1312 | 0.59122 |  |  |  |
| Total | 321 | 69.041 | 1 |  |  |  |  |

**B**

|  | Df | SumsOfSqs | MeanSqs | F.Model | R2 | Pr(>F) |  |
| --- | --- | --- | --- | --- | --- | --- | --- |
| Library | 1 | 0.1807 | 0.18066 | 1.933 | 0.01437 | 0.045 | * |
| Plot | 1 | 0.1578 | 0.15784 | 1.6889 | 0.01256 | 0.082 | . |
| Days | 1 | 2.6921 | 2.69214 | 28.8047 | 0.21415 | 0.001 | *** |
| Cultivar | 1 | 0.1875 | 0.18745 | 2.0057 | 0.01491 | 0.04 | * |
| Days:Cultivar | 1 | 0.1002 | 0.10023 | 1.0724 | 0.00797 | 0.284 |  |
| Residuals | 99 | 9.2527 | 0.09346 | 0.73603 |  |  |  |
| Total | 104 | 12.571 | 1 |  |  |  |  |

**C**

|  | Df | SumsOfSqs | MeanSqs | F.Model | R2 | Pr(>F) |  |
| --- | --- | --- | --- | --- | --- | --- | --- |
| Plot | 1 | 0.2625 | 0.2625 | 1.6905 | 0.01313 | 0.048 | * |
| Days | 1 | 3.5609 | 3.5609 | 22.931 | 0.17807 | 0.001 | *** |
| Cultivar | 1 | 0.2605 | 0.2605 | 1.6773 | 0.01302 | 0.045 | * |
| Days:Cultivar | 1 | 0.2298 | 0.2298 | 1.4801 | 0.01149 | 0.093 | . |
| Residuals | 101 | 15.6839 | 0.1553 | 0.78429 |  |  |  |
| Total | 105 | 19.9976 | 1 |  |  |  |  |

**D**

|  | Df | SumsOfSqs | MeanSqs | F.Model | R2 | Pr(>F) |  |
| --- | --- | --- | --- | --- | --- | --- | --- |
| Library | 1 | 0.1777 | 0.1777 | 1.237 | 0.00833 | 0.193 |  |
| Plot | 1 | 0.1965 | 0.1965 | 1.368 | 0.00921 | 0.149 |  |
| Days | 1 | 5.3979 | 5.3979 | 37.576 | 0.25306 | 0.001 | *** |
| Cultivar | 1 | 0.2834 | 0.2834 | 1.973 | 0.01329 | 0.041 | * |
| Days:Cultivar | 1 | 0.1915 | 0.1915 | 1.333 | 0.00898 | 0.158 |  |
| Residuals | 105 | 15.0836 | 0.1437 | 0.70713 |  |  |  |
| Total | 110 | 21.3307 | 1 |  |  |  |  |

**E**

|  | Df | SumsOfSqs | MeanSqs | F.Model | R2 | Pr(>F) |  |
| --- | --- | --- | --- | --- | --- | --- | --- |
| Days | 1 | 0.4442 | 0.44423 | 3.5364 | 0.06396 | 0.001 | *** |
| Cultivar | 1 | 0.1296 | 0.12959 | 1.0316 | 0.01866 | 0.392 |  |
| Days:Cultivar | 1 | 0.0907 | 0.09072 | 0.7222 | 0.01306 | 0.81 |  |
| Residuals | 50 | 6.2809 | 0.12562 | 0.90432 |  |  |  |
| Total | 53 | 6.9454 | 1 |  |  |  |  |

**F**

|  | Df | Sum Sq | Mean Sq | F value | Pr(>F) |  |
| --- | --- | --- | --- | --- | --- | --- |
| Timepoint | 6 | 34.39 | 5.73 | 3.645 | 0.001644 | ** |
| Cultivar | 1 | 348.62 | 348.62 | 221.7074 | < 2.2e-16 | *** |
| Timepoint:Cultivar | 6 | 65.4 | 10.9 | 6.9323 | 6.35E-07 | *** |
| Residuals | 308 | 484.31 | 1.57 |  |  |  |
