# Supplementary material for: Comparative Analysis of Root Microbiomes of Rice Cultivars with High and Low Methane Emissions Reveals Differences in Abundance of Methanogenic Archaea and Putative Upstream Fermenters
Source: mSystems. 2020 Feb 18;5(1):e00897-19. doi: 10.1128/mSystems.00897-19 (PMC7029222; doi:10.1128/mSystems.00897-19)
Supplement: TABLE S2 [file mSystems.00897-19-st002.docx]

| Compartment | trait | term | sumsq | meansq | statistic | p.value | p.adjusted |
| --- | --- | --- | --- | --- | --- | --- | --- |
| Rhizosphere | chemoheterotrophy | Cultivar | 25740.82983 | 25740.82983 | 1.216132805 | 0.273028846 | 0.344878542 |
| Rhizosphere | dark_hydrogen_oxidation | Cultivar | 1571.945049 | 1571.945049 | 8.168011739 | 0.005284406 | 0.01409175 |
| Rhizosphere | fermentation | Cultivar | 2331.558965 | 2331.558965 | 2.96139415 | 0.088672838 | 0.15201058 |
| Rhizosphere | iron_respiration | Cultivar | 794.9878708 | 794.9878708 | 1.296221112 | 0.257893073 | 0.343857431 |
| Rhizosphere | methanogenesis | Cultivar | 499.6093564 | 499.6093564 | 21.71614785 | 1.08E-05 | 0.000129196 |
| Rhizosphere | methanotrophy | Cultivar | 16.01623472 | 16.01623472 | 0.403047243 | 0.527112722 | 0.60241454 |
| Rhizosphere | reductive_acetogenesis | Cultivar | 358.6525583 | 358.6525583 | 8.80592199 | 0.003836153 | 0.011508458 |
| Rhizosphere | sulfate_respiration | Cultivar | 5413.451985 | 5413.451985 | 2.291834162 | 0.133521753 | 0.213634805 |
| Endosphere | chemoheterotrophy | Cultivar | 159061.7169 | 159061.7169 | 10.18190418 | 0.001911297 | 0.009463255 |
| Endosphere | dark_hydrogen_oxidation | Cultivar | 1125.698579 | 1125.698579 | 6.144526592 | 0.014908187 | 0.032526954 |
| Endosphere | fermentation | Cultivar | 22125.76477 | 22125.76477 | 22.28281154 | 7.92E-06 | 0.000129196 |
| Endosphere | iron_respiration | Cultivar | 6198.683996 | 6198.683996 | 10.11866449 | 0.001971512 | 0.009463255 |
| Endosphere | methanogenesis | Cultivar | 96.46063826 | 96.46063826 | 6.929120846 | 0.009867786 | 0.023682686 |
| Endosphere | methanotrophy | Cultivar | 189.5657967 | 189.5657967 | 1.803694407 | 0.182400546 | 0.273600819 |
| Endosphere | reductive_acetogenesis | Cultivar | 1201.651256 | 1201.651256 | 16.09269109 | 0.000118675 | 0.000949403 |
| Endosphere | sulfate_respiration | Cultivar | 30736.24631 | 30736.24631 | 8.927810598 | 0.003557407 | 0.011508458 |
| Rhizoplane | chemoheterotrophy | Cultivar | 703.2039912 | 703.2039912 | 0.05948514 | 0.807854619 | 0.807854619 |
| Rhizoplane | dark_hydrogen_oxidation | Cultivar | 186.1880534 | 186.1880534 | 1.532597089 | 0.218873034 | 0.308997225 |
| Rhizoplane | fermentation | Cultivar | 2796.218024 | 2796.218024 | 5.26361027 | 0.02405128 | 0.048102561 |
| Rhizoplane | iron_respiration | Cultivar | 98.30765908 | 98.30765908 | 0.259044991 | 6.12E-01 | 0.665550326 |
| Rhizoplane | methanogenesis | Cultivar | 52.52191331 | 52.52191331 | 4.919533607 | 0.029016258 | 0.053568476 |
| Rhizoplane | methanotrophy | Cultivar | 16.48617431 | 16.48617431 | 0.223083262 | 0.637819062 | 0.665550326 |
| Rhizoplane | reductive_acetogenesis | Cultivar | 612.1502755 | 612.1502755 | 9.109539826 | 0.003289417 | 0.011508458 |
| Rhizoplane | sulfate_respiration | Cultivar | 1451.093773 | 1451.093773 | 0.706644687 | 0.402739422 | 0.483287306 |
