# Supplementary figures and images for: Comparative Analysis of Root Microbiomes of Rice Cultivars with High and Low Methane Emissions Reveals Differences in Abundance of Methanogenic Archaea and Putative Upstream Fermenters
Source: mSystems. 2020 Feb 18;5(1):e00897-19. doi: 10.1128/mSystems.00897-19 (PMC7029222; doi:10.1128/mSystems.00897-19)

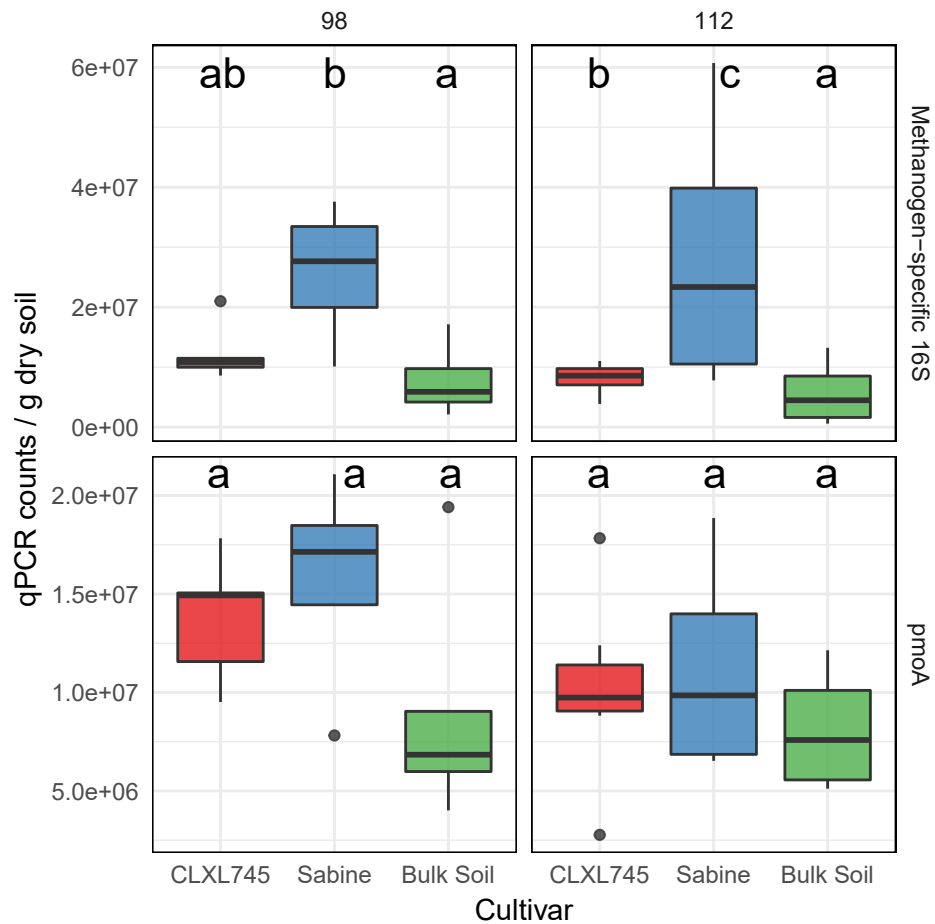

Supplement: FIG S2 [file mSystems.00897-19-sf002.pdf]

**A**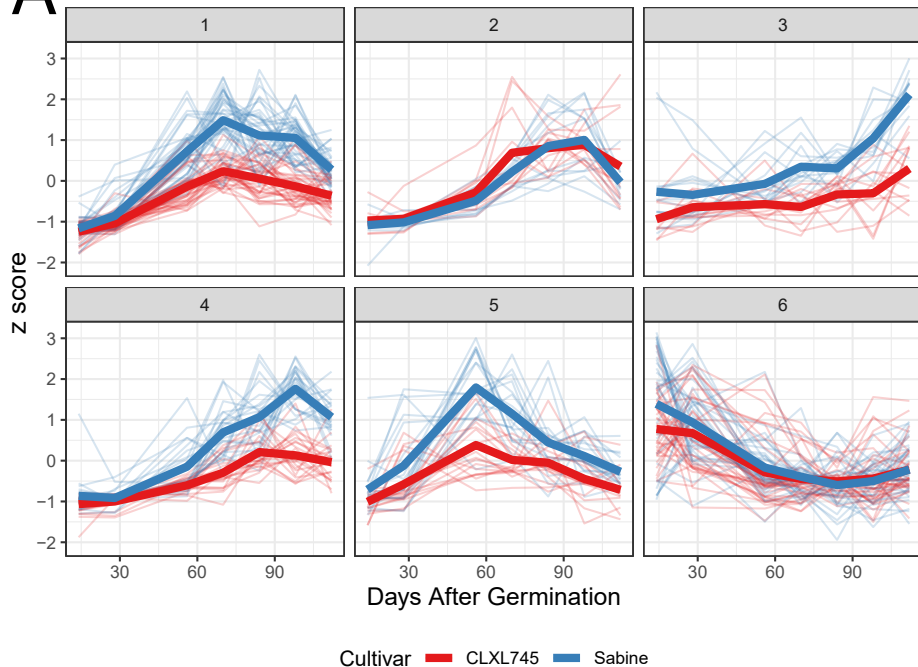**B**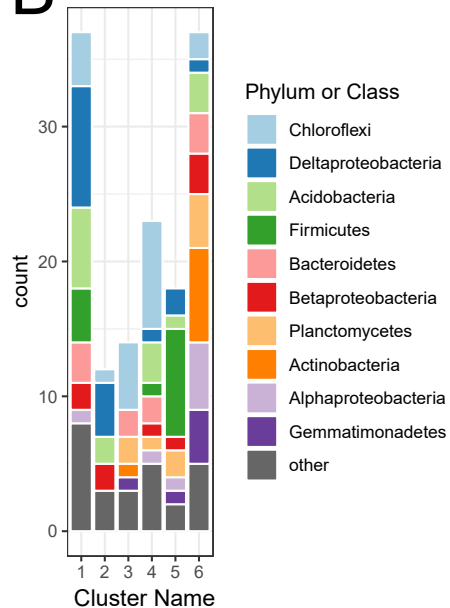

Supplement: FIG S4 [file mSystems.00897-19-sf004.pdf]
