# Supplementary material for: Comparative Analysis of Root Microbiomes of Rice Cultivars with High and Low Methane Emissions Reveals Differences in Abundance of Methanogenic Archaea and Putative Upstream Fermenters
Source: mSystems. 2020 Feb 18;5(1):e00897-19. doi: 10.1128/mSystems.00897-19 (PMC7029222; doi:10.1128/mSystems.00897-19)
Supplement: FIG S3 [file mSystems.00897-19-sf003.pdf]

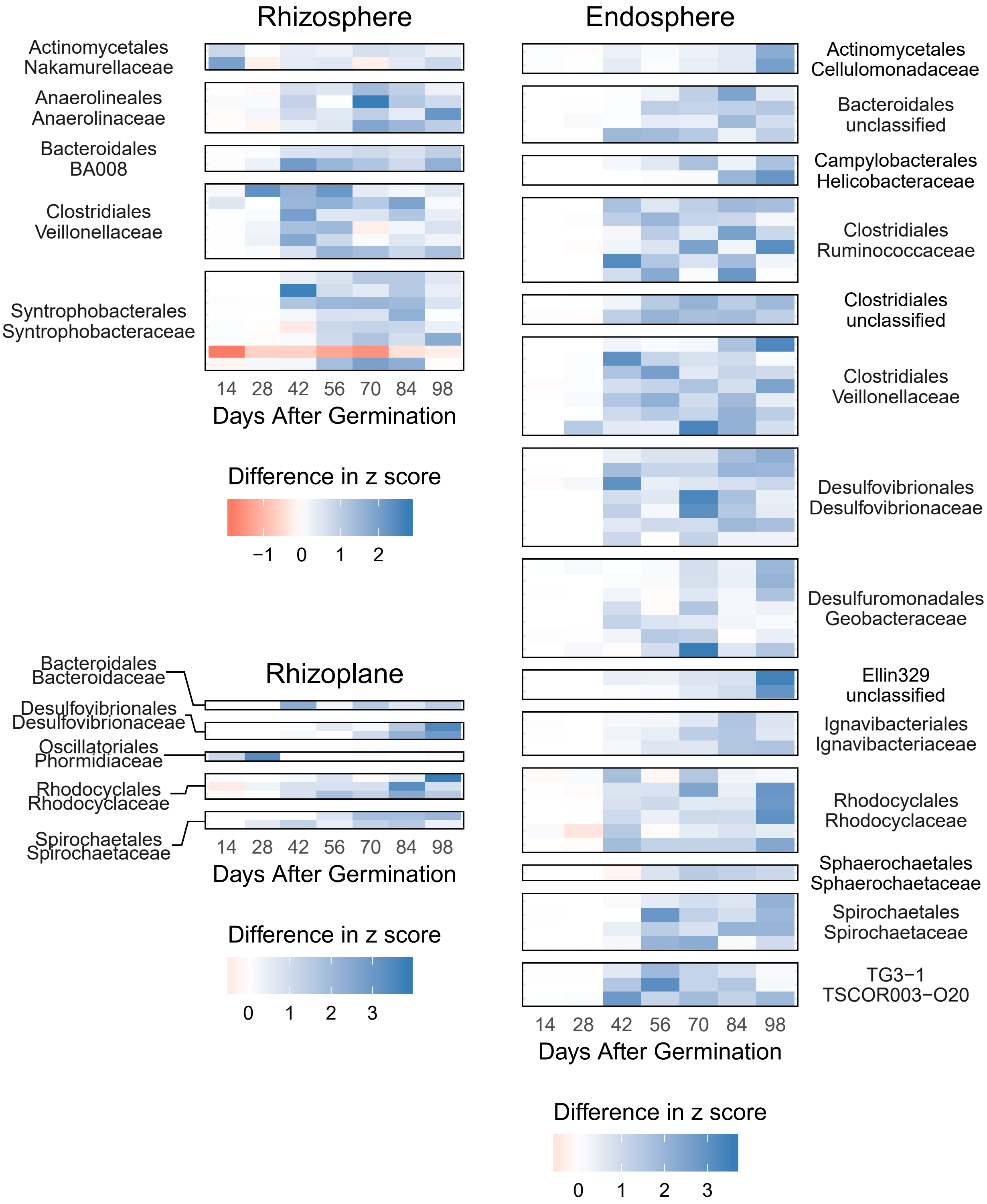

Rhizosphere
Actinomycetales Nakamurellaceae
Anaerolineales Anaerolinaceae
Bacteroidales BA008
Clostridiales Veillonellaceae
Syntrophobacterales Syntrophobacteraceae
14 28 42 56 70 84 98
Days After Germination
Difference in z score
−1 0 1 2
Rhizoplane
Bacteroidales Bacteroidaceae
Desulfovibrionales Desulfovibrionaceae
Oscillatoriales Phormidiaceae
Rhodocyclales Rhodocyclaceae
Spirochaetales Spirochaetaceae
14 28 42 56 70 84 98
Days After Germination
Difference in z score
0 1 2 3
Endosphere
Actinomycetales Cellulomonadaceae
Bacteroidales unclassified
Campylobacterales Helicobacteraceae
Clostridiales Ruminococcaceae
Clostridiales unclassified
Clostridiales Veillonellaceae
Desulfovibrionales Desulfovibrionaceae
Desulfuromonadales Geobacteraceae
Ellin329 unclassified
Ignavibacteriales Ignavibacteriaceae
Rhodocyclales Rhodocyclaceae
Sphaerochaetales Sphaerochaetaceae
Spirochaetales Spirochaetaceae
TG3−1 TSCOR003−O20
14 28 42 56 70 84 98
Days After Germination
Difference in z score
0 1 2 3
