# Supplementary material for: Comparative Analysis of Root Microbiomes of Rice Cultivars with High and Low Methane Emissions Reveals Differences in Abundance of Methanogenic Archaea and Putative Upstream Fermenters
Source: mSystems. 2020 Feb 18;5(1):e00897-19. doi: 10.1128/mSystems.00897-19 (PMC7029222; doi:10.1128/mSystems.00897-19)
Supplement: TABLE S3 [file mSystems.00897-19-st003.docx]

**A**

|  | Df | Sum Sq | Mean Sq | F value | Pr(>F) |
| --- | --- | --- | --- | --- | --- |
| Timepoint | 3 | 0.044291 | 0.014764 | 5.0349 | 0.02217 |
| Cultivar | 1 | 0.000494 | 0.000494 | 0.1685 | 0.6901 |
| Timepoint:Cultivar | 3 | 0.012924 | 0.004308 | 1.4692 | 0.28139 |
| Residuals | 10 | 0.029322 | 0.002932 |  |  |

**B**

|  | Df | Sum Sq | Mean Sq | F value | Pr(>F) |
| --- | --- | --- | --- | --- | --- |
| Timepoint | 3 | 2784.36 | 928.12 | 31.2651 | 6.25E-12 |
| Cultivar | 1 | 445.14 | 445.14 | 14.9954 | 0.000289 |
| Timepoint:Cultivar | 3 | 17.18 | 5.73 | 0.1929 | 0.900793 |
| Residuals | 55 | 1632.7 | 29.69 |  |  |

**C**

| Timepoint | contrast | estimate | SE | df | t.ratio | p.value |
| --- | --- | --- | --- | --- | --- | --- |
| 1 | CLXL745-Sabine | -4.099476 | 2.724221 | 55 | -1.505 | 0.1381 |
| 2 | CLXL745-Sabine | -4.708275 | 2.724221 | 55 | -1.728 | 0.0895 |
| 3 | CLXL745-Sabine | -5.642603 | 2.819837 | 55 | -2.001 | 0.0503 |
| 4 | CLXL745-Sabine | -6.847717 | 2.724221 | 55 | -2.514 | 0.0149 |
